# City Environment and Occurrence of Neural Autoantibodies in Psychiatric Patients

**DOI:** 10.3389/fpsyt.2022.937620

**Published:** 2022-07-07

**Authors:** Niels Hansen, Aaron Levin Juhl, Insa Maria Grenzer, Bianca Teegen, Jens Wiltfang, Dirk Fitzner

**Affiliations:** ^1^Department of Psychiatry and Psychotherapy, University of Göttingen, Göttingen, Germany; ^2^Translational Psychoneuroscience, University of Göttingen, Göttingen, Germany; ^3^Euroimmun Reference Laboratory, Lübeck, Germany; ^4^German Center for Neurodegenerative Diseases (DZNE), Göttingen, Germany; ^5^Department of Medical Sciences, Neurosciences and Signaling Group, Institute of Biomedicine, University of Aveiro, Aveiro, Portugal; ^6^Department of Neurology, University Medical Center Göttingen, Göttingen, Germany

**Keywords:** urban environment, major city, autoimmunity, psychiatry, neuronal autoantibodies

## Abstract

**Background:**

City living might lead to a higher risk of psychiatric disease, but to date there is no evidence of any correlation between an urban environment and the occurrence of neural autoantibodies in psychiatric disease. Our aim is to identify whether the number of patients with and without neural autoantibodies living in diverse rural and urban environments differ.

**Methods:**

We enrolled retrospectively a cohort of 167 psychiatric patients *via* a cross-sectional design from the Department of Psychiatry and Psychotherapy University Medical Center Göttingen and determined serum and/or CSF neural autoantibodies in them. The patients live in the German states of Lower Saxony, Thuringia, and Hessen. Their data were investigated in conjunction with the location of their primary residence. We categorized them into five different categories depending upon their primary residence: one rural and four different urban environments depending on their population numbers.

**Results:**

We identified 36 psychiatric patients with neural autoantibodies, and 131 psychiatric patients with none. In total, 24 psychiatric patients with neural autoantibodies were classified as sharing a possible, probable, or definitive autoimmune origin according to our recently set criteria. We observed as a non-significant trend that more psychiatric patients with neural autoantibodies and a probable or definitive autoimmune origin (45.8%) live in a major city with over 100,000 inhabitants than do psychiatric patients presenting no evidence of autoantibodies (26.4%). However, we identified no relevant differences between (1) psychiatric patients with and without neural autoantibodies or between (2) psychiatric patients with a possible, probable, or definitive autoimmune origin and those without such autoantibodies in relation to the diverse rural and urban environmental settings.

**Conclusion:**

The inherently different aspects of rural and urban environments do not appear to be relevant in determining the frequency of neural autoantibodies in psychiatric patients in Lower Saxony, Thuringia, and Hessen in Germany. Furthermore, large-scale studies involving other states across Germany should be conducted to exclude any regional differences and to examine the tendency of a higher frequency in large cities of autoimmune-mediated psychiatric syndromes.

## Introduction

Psychiatric diseases have multifactorial causes. Genetic and environmental factors interact together with the patient’s individual life events that might result in the manifestation of psychiatric symptoms and potentially culminate in a psychiatric disorder. A recent investigation in a large biobank cohort of 385,793 participants in the United Kingdom showed that a subject’s primary residence can be determined by their genetic risk of psychiatric disease (1). Their study indicates that a subject’s genes are influenced by their environment, which in turn determine an individual’s risk for psychiatric disease. Thus, whether the location of someone’s primary residence, being urban or rural, raises that person’s risk for psychiatric disease depends on their specific genes. In other words, the manifestation of psychiatric disease in its specific urban or rural environment is to some extent genetically determined. Beyond the role that genes play, there is ample evidence that humans react to stress differently according to environmental conditions. The amygdala is a brain structure involved in processing stress. Another study showed that the amygdalas of humans living in an urban environment are more active (2), inducing elevated amygdala-related stress processing in humans in an urban environment. And it is not just the amygdala that is known to be involved in such stress processing in humans in an urban environment, as the same working group (2) demonstrated that social stress processing within the perigenual anterior cingulate cortex is also associated with growing up in a city. The amygdala is known to be involved in the autoantibody-mediated disease of the central nervous system, such as autoimmune encephalitis (3). Thus, it is tempting to speculate that an autoantibody-driven inflammation of the amygdala would be much more active in an urban area through stress processing combined with inflammation, which would thereby facilitate the induction or exaggeration of emotional states in psychiatric patients leading to hospital admission. These studies demonstrate that living in an urban environment can alter the conditions that induce psychiatric symptoms and even psychiatric disease. However, we do not yet know whether the neural autoantibodies that play a growing role in specific subtypes of the psychiatric disease are also influenced by such environmental factors. Several recently developed criteria serve to classify psychiatric patients with neural autoantibodies into those that might have an autoimmune origin (4) that often differ from non-organic psychotic disorders with other pathomechanisms responsible for generating disease. There are other criteria to define autoimmune encephalitis (5) in which psychiatric symptoms are potentially a relevant component or even the main phenotype, as described in a study by Endres et al. (6). A recent study (7) demonstrated a higher seroprevalence of glutamic acid decarboxylase 65 (GAD65) autoantibodies in African humans in a rural environment. Rural and city environments in Africa and Europe are extremely diverse (due to the degree of industrialization, cultural factors, etc.), which makes such research findings difficult to apply to industrial environmental conditions in more advanced industrial cultures. GAD65 antibodies are more frequently detected in rural Ghana populations; they are mainly influenced by the presence of fever, and the patient’s medical history, i.e., fevers and higher liver enzyme concentrations (7). Reasons for these factors are unknown, but might be attributable to less available medical care in rural than in urban Ghana populations. These conditions are absent in Europe and other industrialized cultures where adequate medical care is available in both rural and urban locations. Nevertheless, the serum prevalence of neural autoantibodies in psychiatric patients might be determined by environmental factors, such as specific population-based viruses, which spread more extensively in cities, and eventually might cause neural autoantibodies to be produced as an automatic autoimmune process following severe viral infection, as depicted in a study (8) with increased neural autoantibodies in patients with COVID-19 with neuropsychiatric symptoms. Several non-pathogenic microorganisms that regularly induce immune reactions might lead to less autoimmune disease, as the immune system is better trained to distinguish pathogenic from non-pathogenic antigens. Based on these hypothetical reflections, our hypothesis is—in an urban environment—to detect a higher frequency of psychiatric patients presenting neural autoantibodies than psychiatric patients without neural autoantibodies. Our investigation’s objective is to assess the occurrence vs. non-occurrence of autoantibodies in psychiatric patients in urban and rural populations.

## Materials and Methods

We retrospectively enrolled a study cohort of 167 psychiatric patients with diagnosis classes from F00–F69 according to the International Statistical Classification of Diseases and Related Health Problems, 10th Revision (ICD10 system) from the Department of Psychiatry and Psychotherapy University Medical Center Göttingen between 2017 and 2020 in which we determined serum and/or cerebrospinal (CSF) neural autoantibodies analyzed in the Euroimmun laboratory in Lübeck. We screened these patients for autoantibodies for differential-diagnostic reasons. Our patient cohort’s (*n* = 167 patients) data was sourced from patient files exclusively from the in- and outpatient Department of Psychiatry University Medical Center Göttingen (psychiatric patients with different diagnoses) in 2017–2020 whose neural autoantibodies we had determined for differential diagnostic reasons. Their serum and/or CSF neural autoantibodies were not routinely assessed, as this was part of taking a differential diagnostic approach when we theorized a possible organic reason for psychiatric symptoms, i.e., by excluding an inflammatory or autoinflammatory central nervous system disease. It was the clinical presentation of all patients that prompted us to undertake an extensive differential diagnosis before making a diagnosis at their admission to our hospital, which would have entailed the examination of biological samples and seeking serum or CSF autoantibodies (see below for more details). The diagnoses we made clinically are in line with the ICD10 system ranging from F00–F09, F10–F19, F20–F29, F30–F39, F40–F49, to F60–F69. Our study was retrospective in nature and is characterized by a cross-sectional design in determining neural autoantibodies in conjunction with the primary residency of patients. COVID-19 infection was excluded in the investigated patients as it would have affected the profile of neural autoantibodies. The following autoantibodies in patients were determined as a standard panel in serum and/or CSF *via* Euroline immunoblots and cell-based assays [autoantibodies against intracellular autoantibodies: amphiphysin, CV2, GAD65, HuD, Ma1/Ma2, neurochondrin (NC), Ri, TR, Yo, and Zic4; autoantibodies against cell surface targets: α-amino-3-hydroxy-5-methyl-4-isoxazolepropionic acid receptors 1/2 (AMPAR1/2), Aquaporin 4, contactin-associated protein 2 (CASPR2), dipeptidyl-peptidase-like 6 protein (DPPX), gamma-aminobutyric acid B1/2 receptor (GABAB1/2R), glutamic acid decarboxylase (GAD65), Leucin Rich Glioma Inactivated Protein 1 (LGI1), N-methyl-D-aspartate receptor (NMDAR)]. This standard panel of autoantibodies was determined in all the patients by assessing the most relevant neural autoantibodies against membrane-surface and intracellular antigens, although it would make sense to assess only specific autoantibodies in association with specific psychiatric diseases, i.e., anti-basal ganglia antibodies in obsessive-compulsive disorder (9) or neural cell adhesion molecule 1 antibodies in patients with schizophrenia (10) to limit costs. However, there is no evidence to date of clear types of autoantibodies that are specific to only one psychiatric condition (4). Most neural autoantibodies can coincide with several specific psychiatric diseases and are not specific to any one disease. All of our study patients lived in the German states of Lower Saxony, Thuringia, and Hessen. Their data were investigated in conjunction with the location of their primary residence. The urban and rural populations were differentiated when we were determining their neural autoantibodies. Primary residency refers to the patients’ main living location while their neural autoantibodies were being assessed. Blood and urine parameters were not systematically assessed in patients. We categorized them into five different categories depending on their primary residence: one rural and four different urban environments depending on their population numbers. We considered a town population of less than 2,000 as a rural environment. In total, 2,000–5,000 inhabitants were considered a rural town, 5,000–20,000 inhabitants were classified as a small city, 20,000–100,000 as a medium-sized city, and over 100,000 inhabitants a large city. The population numbers in the rural and urban environments were obtained from the Landesamt für Statistik Niedersachsen (11) from the published population census. In the second step, we grouped the patients into two categories by merging the aforementioned categories according to the following procedure. We categorized an environment as rural by adding the rural and rural town environmental dimensions comprising less than 5,000 inhabitants. More than 5,000 inhabitants were then classified as an urban environment (having merged small, medium, and large city conditions). Furthermore, psychopathology was assessed by relying on patient records and was measured by the AMDP system (12). We relied on the AMDP classification system to group symptoms into syndromes. Electroencephalographic (EEG), magnetic resonance imaging (MRI), and CSF analysis data were taken from our patient database. CSF data were retrieved from lumbar puncture samples taking a differential diagnostic approach. CSF parameters, such as cell count, a blood–brain barrier disturbance, intrathecal IgG synthesis, and neurodegeneration markers, such as *t*-tau, phosphorylated tau protein 181 (p-tau181), amyloid-ß 42 (Aß42), amyloid-ß 40 (Aß40) and ratio of Aß42/40, were assessed in the Neurochemistry CSF Laboratory of the Department of Neurology, University Medical Center Göttingen. Our study followed the tenets of the Declaration of Helsinki and was approved by our local ethics committee.

### Statistical Approach

We used Fisher’s exact test to compare the frequencies of (1) psychiatric patients with and without serum and CSF neural autoantibodies, (2) psychiatric patients with probable or definitive autoimmune-based psychiatric syndrome vs. those psychiatric patients without neural autoantibodies and (3) psychiatric patients with CSF autoantibodies and those with no autoantibodies at all. The age, number of inhabitants, and CSF parameters, such as cell count, protein content, and neurodegeneration markers, were assessed by paired Student’s *t*-tests with a later performed Bonferroni correction for multiple testing. A *p*-level of *p* < 0.05 was considered as a relevant difference between groups.

## Results

### Description of Psychiatric Patients’ Cohorts

**FIGURE 1:**
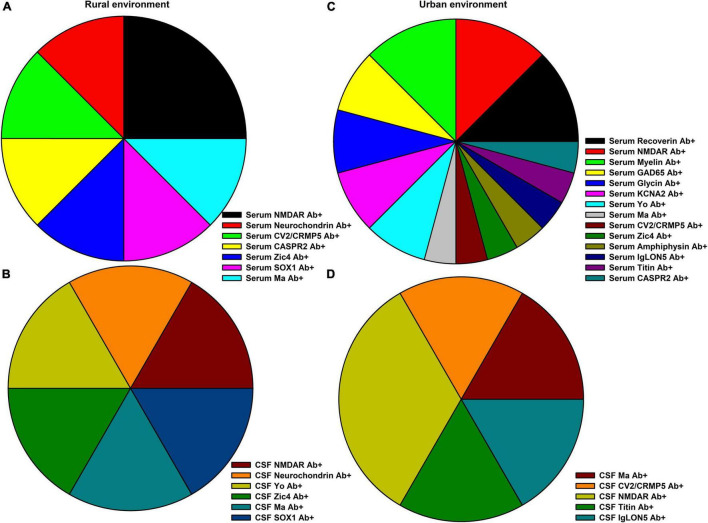
Neural autoantibody spectrum in rural and urban environments. **(A)** The spectrum of serum and **(B)** of cerebrospinal fluid (CSF) neural autoantibody occurrence in psychiatric patients (pAb+) in a rural environment are depicted, whereas **(C)** the spectrum of serum and **(D)** of CSF neural autoantibodies in an urban environment are shown.

In our cohort (*n* = 167 psychiatric patients), we identified 36 patients with serum or CSF autoantibodies (pAb +). In total, 131 psychiatric patients had no autoantibodies (pAb-). These patients were not infected with COVID-19 (an exclusion criterion of the study) as mentioned in the methods section. No differences emerged between the frequency of taking certain psychopharmacologic drugs between pAb + and pAb- groups [pAb + : antidepressant drugs in 15/36 (42%), antipsychotic drugs in 7/36 (19.4%), mood-stabilizing drugs in 2/36 (5.5%), anxiolytic drugs in 1/36 (2.8%), hypnotic drugs in 1/36 (2.8%), and in 1/36 (1.5%) antidementia drugs; pAb-: antidepressant drugs in 57/131 (43.6%), antipsychotic drugs in 37/131 (28.6%), mood-stabilizing drugs in 9/131 (6.9%), mood-stabilizing drugs in 19/131(14.5%), and also anxiolytic and antidementia drugs in 2/131 (1.5%)]. According to our criteria (4), of the psychiatric patients presenting neural autoantibodies, 13/36 (36%) had a probable and 11/36 (31%) a definitive autoimmune-based psychiatric syndrome (the former and latter were defined as paAb + patients). Applying the Graus criteria (5) yielded results resembling the application of the Hansen criteria (4), as according to the Graus et al. criteria, 11/36 (31%) patients had possible autoimmune encephalitis and 12/36 (33%) definitive autoimmune encephalitis. In total 12 of 36 (33%) of the pAb + patients had CSF autoantibodies and were classified as pCSFAb + patients. Figure 1 depicts the spectrum of neural serum and CSF autoantibodies in pAb + patients in urban and rural environments. The distribution of psychiatric syndromes in pAb + and pAb- patients is depicted in Table 1. The pAb + and pAb- patients in an urban environment as primary residency did not differ in the terms of patient’s age, gender, number of city inhabitants, CSF data, MRI data, and EEG data (Table 1). Furthermore, the pAb + and pAb- patients in a rural environment as primary residency did not differ in terms of patient age, gender, CSF data, MRI data, or EEG data (Table 1). However, we observed (as a non-significant trend after correction for multiple testing) that pAb + patients were older than the pAb- patients living primarily in an urban environment. Moreover, we detected (as a non-significant trend after correction for multiple testing) fewer paranoid hallucinatory syndromes in pAb + compared with pAb- patients living primarily in an urban environment. The occurrence of psychiatric diagnosis according to ICD10 of pAb + and pAb- patients did not differ whether they lived primarily in a rural or urban environment (Table 1). Furthermore, the autoimmune diseases as conditions for autoimmunity did not differ between pAb + and pAb- patients in an urban vs. a rural setting (Table 1). Note that patients with and without autoantibodies were not all psychiatric patients. About 42% of pAb + and 47% of pAb- patients with a primary urban residence and 43% of pAb + and 42% pAb- patients with a primary rural residence had neurological deficits. The neurological deficits did not differ between groups living in an urban versus rural environment (Table 1). The frequency of tumors potentially indicating a paraneoplastic condition coinciding with neural autoantibodies does not differ between patients with and without autoantibodies living in an urban vs. rural environment (Table 1).

**TABLE 1:** Demographic, clinical, and laboratory data of patient groups.

	Rural environment PAB + (*N* = 7)	Rural environment PAB- (*N* = 19)	Statistics rural PAB + VS PAB-	Urban environment PAB + (*N* = 29)	Urban environment PAB- (*N* = 110)	Statistics urban PAB + VS PAB-
Age Years	57.6 ± 13.3	66 ± 14	0.58	62.6 ± 16	54 ± 18	<0.05*
Gender (Female)	3/7 (43%)	13/19 (68%)	0.37	13/29 (45%)	58/110 (53%)	0.53
Inhabitants	2406 ± 1363	2529 ± 1761	0.09	65937 ± 50918	59386 ± 82122	0.46
Psychiatric syndromes						
Apathic	1/7 (14%)	3/19 (16%)	1	3/29 (10%)	11/116 (9.5%)	1
Depressive	4/7 (57%)	14/19 (59%)	0.64	17/29 (59%)	62/116 (53%)	0.68
Hostility	0/7 (0%)	1/19 (5%)	1	1/29 (3%)	6/106 (6%)	1
Maniforme	0/7 (0%)	0/19 (0%)	1	0/29 (0%)	1/106 (0.9%)	1
Neurologic	0/7 (0%)	3/19 (16%)	0.54	3/29 (10%)	16/106 (15%)	0.76
Obessive-Compulsive	0/7 (0%)	0/19 (0%)	1	0/29 (0%)	8/106 (7,5%)	0.20
Parahallucinatory	2/7 (29%)	6/19 (3.2%)	1	2/29 (7%)	29/106 (27%)	<0.05*
Psychorganic	6/7 (86%)	17/19 (91%)	1	21/29 (72%)	81/106 (77%)	0.63
Vegetative	1/7 (14%)	2/19 (11%)	1	0/29 (0%)	7/106 (7%)	0.34
Psychiatric diagnoses						
F00-F09	3/7 (43%)	8/19 (42%)	1	21/29 (72%)	57/110 (52%)	0.06
F10-F19	0/7 (0%)	1/19 (5%)	1	0/29 (0%)	2/110 (2%)	0.34
F20-F29	2/7 (29%)	4/19 (21%)	0.64	2/29 (7%)	19/110 (18%)	0.24
F30-39	2/7 (29%)	4/19 (21%)	0.64	4/29 (21%)	27/110 (25%)	0.31
F40-49	0/7 (0%)	0/19 (0%)	1	2/29 (7%)	2/110 (2%)	0.17
F60-69	0/7 (0%)	1/19 (5%)	1	9/29 (31%)	20/110 (18%)	0.19
Autoimmune disease	1/7 (14%)	2/19 (11%)	1	1/29 3%)	2/110 (2%)	0.51
Tumor	1/7 (14%)	7/19 (37%)	0.37	9/29 (31%)	20/110 (18%)	0.17
Neurological deficit	3/7 (43%)	8/19 (42%)	1	12/29 (42%)	52/110 (47%)	0.67
CSF						
Cell count (<5 M G/L)	1 ± 1.2	0.37 ± 0.49	0.21	1.4 ± 2.3	1.7 ± 7.7	0.53
Total protein count (MG/L)	507 ± 124	406 ± 96	0.08	704 ± 222	418 ± 205	0.76
Intrathecal igg synthesis	1/7 (14%)	0/19 (0%)	0.27	3/29 (10%)	11/103 (11%)	1
Blood brain barrier bisturbance	2/7 (29%)	1/19 (5%)	0.59	3/29 (10%)	24/103 (23%)	0.19
T tau protein (<450 PG/ML)	365.7 ± 369	311 ± 444	0.79	456 ± 320	421 ± 339	0.89
P tau 181 (<61 PG/ML)	71 ± 68	66 ± 46	0.88	82 ± 61	69 ± 36	0.19
Aß42 (>450 PG/ML)	1119 ± 552	1071 ± 473	0.80	1081 ± 624	1084 ± 603	0.78
Aß40	11378.5 ± 63333	11342 ± 3823	0.99	11597 ± 6192	11001 ± 5820	0.66
Ratio Aß42/Aß40 (X10: > 0.5)	1.15 ± 0.63	0.92 ± 0.42	0.34	1 ± 0.55	1.01 ± 0.56	0.78
MRI						
Generalized atrophy	0/5 (0%)	10/18 (55%)	< 0.05*	9/25 (36%)	30/95 (32%)	0.81
	Rural environment PAB + (*N* = 7)	Rural environment PAB- (*N* = 19)	Statistics rural PAB + VS PAB-	Urban environment PAB + (*N* = 29)	Urban environment PAB- (*N* = 110)	Statistics urban PAB + VS PAB-
Focal atrophy	3/5 (60%)	6/18 (33%)	0.34	10/25 (40%)	21/95 (22%)	0.08
Hippocampal atrophy	0/5 (0%)	1/18 (6%)	1	1/25 (4%)	5/95 (5%)	1
EEG						
Temporal focal slowing	3/5 (60%)	5/9 (55%)	1	6/17 (35%)	25/80 (31%)	0.78
Temporal epileptic potentials	0/5 (0%)	0/9 (0%)	1	1/17 (6%)	2/80 (40%)	0.44
Non-temporal focal slowing	3/5 (60%)	5/9 (55%)	1	5/17 (29%)	23/80 (29%)	1
Non-temporal epileptic potentials	0/5 (0%)	1/9 (11%)	1	0/17 (0%)	1/80 (1%)	1

Aß42, ß-amyloid 42, Aß40, ß-amyloid 40, CSF, cerebrospinal fluid, EEG, electroencephalography, mg/L, milligram per liter, MRI, magnetic resonance imaging, P tau protein 181, phosphorylated tau protein 181, pAb +, psychiatric patients with neural autoantibodies, pAb-, psychiatric patients without neural autoantibodies, pg/ml, pikogram/milliliter, ratio Aß42/40, ratio ß-amyloid 42/ß-amyloid 40, μg/L, mikrogram per liter, Statistics: *p < 0.05 Fisher?s exact test, not significant in a later performed correction for multiple testing.

### Psychiatric Patients Presenting Neural Autoantibodies in Urban and Rural Environments

**FIGURE 2:**
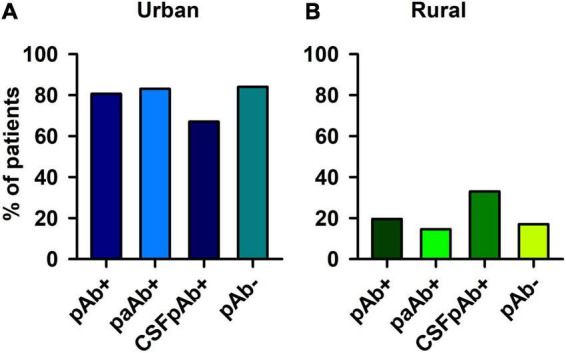
Psychiatric patients with and without neural autoantibodies in urban and rural environment. Psychiatric patients presenting neural autoantibodies (pAb+), psychiatric patients with neural autoantibodies of a probable or definitive autoimmune origin (paAb+) and psychiatric patients with CSF neural autoantibodies (CSFpAb +) did not differ each from psychiatric patients without neural autoantibodies (pAb-) in an **(A)** urban and **(B)** rural environment.

**FIGURE 3:**
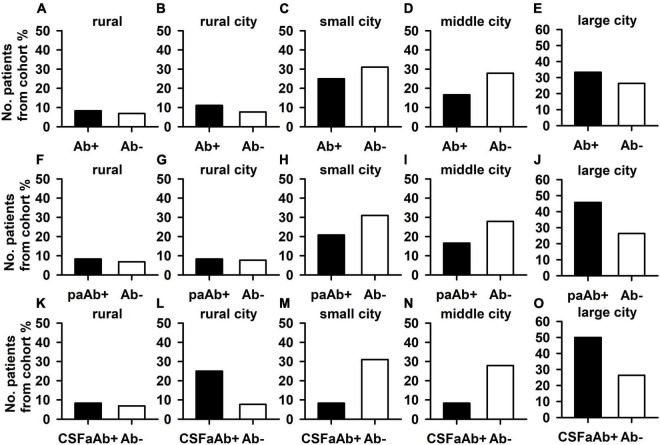
Psychiatric patients with and without neural autoantibodies in different dimensions of an urban and rural environment. The percentages of psychiatric patients with neural autoantibodies (Ab+) did not differ from those without neural autoantibodies in all dimensions: rural (less than 2,000 inhabitants) **(A)**, rural town (2,000–5,000 inhabitants) **(B)**, small city (5,000–20,000 inhabitants) **(C)**, middle city (20,000–100,000 inhabitants as a medium-sized city), **(D)** and large city (over 100,000 inhabitants a large city) **(E)**. Furthermore, the percentage of psychiatric patients with neural autoantibodies and a probable or definitive autoimmune origin (paAb+) did not differ from the percentage of those without neural autoantibodies in all dimensions: rural **(F)**, rural town **(G)**, small city **(H)**, middle city **(I)**, and large city **(J)**. Finally, also the percentages of psychiatric patients with CSF neural autoantibodies (CSFaAb+) did not differ from those of patients without neural autoantibodies in all dimensions: rural **(K)**, rural town **(L)**, small city **(M)**, middle city **(N)**, and large city **(O)**. Ab+, neural autoantibody positive, Ab-, neural autoantibody negative.

The frequency of pAb + patients living in an urban environment did not differ from the frequency of pAb- patients living in an urban environment (Figure 2A). Furthermore, the number of pAb- patients living primarily in an urban environment did not differ from the number of pAb- patients doing so (Figure 2B). If the autoimmune origin is considered in particular, also paAb + patients did not differ in their frequency of living in an urban or rural environment compared with pAb- patients (Figures 2A,B). In addition, CSFpAb + did not differ in their frequency in urban or rural conditions from pAb- patients (Figures 2A,B). If the rural and urban environment situations are regarded in subdomains, no significant differences emerged between the frequency of patients living in a rural environment, rural cities, small cities, medium cities, and big cities in pAb + vs. pAb- patients (Figure 3). The frequency of patients living in a rural environment, rural cities, small cities, medium cities, and big cities in paAb + vs. pAb- patients did not differ (Figure 3). However, we noted a non-significant trend in big cities, where more pAb + (45.8%) than pAb- (26.3%) live, but this was an insignificant trend (Figure 3, Fisher’s exact test, *p* = 0.08). Furthermore, we detected no difference in the frequency distribution when investigating only patients with pCSFAb + compared with pAb- patients (Figure 3). Interestingly, as with pAb + patients, we observed a potentially relevant trend, namely that the frequency of pCSFAb + patients (50%) living in big cities is higher than that of pAb- patients (31%) living in big cities (Figure 3, Fisher’s exact test, *p* = 0.20).

## Discussion

Our findings indicate that autoantibody-associated psychiatric disease is not more frequent than psychiatric disease without antibodies in rural vs. urban environments. These findings do not contradict studies that detected a higher frequency of psychiatric disease in an urban environment, as the focus of this study is the frequency of neural autoantibodies in psychiatric patients, and not a psychiatric disease *per se*. Nevertheless, we identified two non-significant trends in our psychiatric patient cohort that might reveal a direction to follow in larger studies with more homogenous psychiatric patient cohorts regarding diagnosis: in a large city environment, the frequencies of neural autoantibody-associated psychiatric syndromes, and the CSF neural autoantibodies associated with the psychiatric disease are higher than they are in autoantibody-negative psychiatric patients. This trend is supported by our hypothesis that neural autoantibodies and psychiatric disease coincide more often in cities compared with psychiatric disease alone. Due to the lack of significance, our hypothesis of a higher frequency of psychiatric patients presenting neural autoantibodies compared with those without neural autoantibodies could not be confirmed.

Nevertheless, one potential explanation for the trend we observed is that viral stimuli may later affect the production of neural autoantibodies. A further explanation for the observed trend is that autoimmune diseases can be triggered by environmental conditions, such as air pollution. Air pollution can trigger increased proinflammation characterized by higher proinflammatory cytokine levels and a switch of T-cells resulting in a higher Th-17 expression, as shown in a study of the autoimmune disorder rheumatoid arthritis (13). Another possibility is that the social environment in cities might affect the development of serum autoantibodies in diabetes, which is observed more often in blue- than white-collar city regions (14). These studies provide various possible explanations for the trend we observed. More investigation is needed in large cohorts in different regions and varying social-environmental contexts measuring the incidences of diverse viruses to investigate this tendency, which is even more pronounced in psychiatric patients with an autoimmune-based psychiatric syndrome or presenting CSF neural autoantibodies. Exposure to many non-pathogenic environmental microorganisms, such as those in rural environments could have a protective effect in preventing the development of diabetes type 1, an autoimmune disease (15) as our hypothesis proposes. Furthermore, environmental factors might also have an influence on the associated cancers present in psychiatric patients with autoantibodies, namely, in 31% of those living in a rural and in 14% living in an urban environment. However, we detected no differences in the presence of cancer between patients with and without autoantibodies in a rural vs. urban environment, thus this factor can probably be weighted as a minor factor contributing to our cohort’s findings. Apart from the relationship between psychiatry and autoimmunity, other aspects should be kept in mind, i.e., city living may offer residents more protection from certain psychiatric diseases as Stier et al. recently showed regarding depressive symptoms (16). This protective effect might be also disease-specific as social-environmental activation plays a major role in specific psychiatric diseases, such as depression. Suicidality and self-harm (as other major psychiatric clinical features) are higher in cities than in the countryside in the United Kingdom and Ireland (17).

### Limitations

Our autoantibody-positive patient cohort possesses enormously heterogeneous autoantibodies, all of which could have different effects. We could not examine these diverse effects as our case numbers are too low to draw relevant conclusions. Another study limitation to mention concerns the relevant age difference as a tendency in patient groups with and without neural autoantibodies living in cities as their primary residence. More large-scale studies are therefore necessary to investigate any systematic age effect that might affect our findings. Surprisingly, we have also detected a higher proportion of patients with a paranoid hallucinatory syndrome in the group of psychiatric patients without neural autoantibodies as a tendency, a finding that contradicts previous reports on autoantibody-mediated psychosis in patients (18–21) and animal models (10) and that might depend on cohort size; this result should be replicated in larger cohorts. Another limitation is that we did not systematically screen for any head injuries, none of the patients’ clinical data suggested any trauma. Another caveat is that we also did not search for autoantibodies of systemic autoimmune diseases, such as anti-dsDNA (double-stranded desoxyribonucleic acid), anti-Sm [protein complexed to six species of nuclear U1 ribonucleic acid (RNA)], anti-RNP (protein complexed to U1 RNA), etc., typical antibodies for systemic lupus erythematosus or other autoantibodies related to rheumatologic autoimmune diseases. In addition, as we did not assess prior infections systematically, we cannot claim that these infections are reasons for the later manifestation of autoimmune disease. Furthermore, our psychiatric cohort does not present the neural autoantibodies associated with neurological conditions, such as autoimmune epilepsy, as we had no patients with LGI1 autoantibodies. One would have to investigate this issue in a more homogeneous cohort of psychiatric patients with a similar diagnosis. Another caveat is that we observed a relative probability of autoimmunity (based on the Graus (5) and Hansen (4) criteria) in 64% of patients, but 36% of them presented no clear signs of any pathogenicity from the autoantibodies we detected. We thus cannot rule out that the neural autoantibodies might not be pathogenic at all in those 36% of patients. However, the observed tendency for individuals living in a large city environment to present a higher relative frequency of CSF neural autoantibodies associated with psychiatric disease (thus of highly probable autoimmune genesis) than the relative frequency of autoantibody-negative psychiatric patients should not be ignored even if the proportion of patients with possibly pathogenic neural autoantibodies is disregarded. We, therefore, maintain that our results are interesting enough to be relevant for clinicians if they are confirmed in large-scale studies. Furthermore, to better evaluate neural autoantibodies associated with peripheral inflammation, it would help to determine blood C-reactive protein in a later investigation.

## Conclusion

Whether an environment is urban or rural has no impact on the occurrence of neural autoantibodies in psychiatric patients, as those with and without autoantibodies do not differ in rural and urban environmental constellations. However, there seems to be a tendency to observe more psychiatric patients presenting neural autoantibodies and a psychiatric disorder of potential autoimmune origin in large cities than psychiatric patients who lack autoantibodies. This trend should be further investigated in large-scale trials, as there is research evidence that specific factors in cities accelerate autoimmune processes.

## Data Availability Statement

The raw data supporting the conclusions of this article will be made available by the corresponding author, without undue reservation.

## Ethics Statement

This study involving human participants was reviewed and approved by the Ethics Committee of the University Medical Center Göttingen. Written informed consent for participation was not required for this study in accordance with the national legislation and the institutional requirements.

## Author Contributions

NH wrote the manuscript. AJ and IG performed the data collection. BT did part of the laboratory testing. JW and DF revised the manuscript for important intellectual content. All authors contributed to the article and approved the submitted version.

## Conflict of Interest

The authors declare that the research was conducted in the absence of any commercial or financial relationships that could be construed as a potential conflict of interest.

## Publisher’s Note

All claims expressed in this article are solely those of the authors and do not necessarily represent those of their affiliated organizations, or those of the publisher, the editors and the reviewers. Any product that may be evaluated in this article, or claim that may be made by its manufacturer, is not guaranteed or endorsed by the publisher.
